# The wolf reference genome sequence (*Canis lupus lupus*) and its implications for *Canis spp.* population genomics

**DOI:** 10.1186/s12864-017-3883-3

**Published:** 2017-06-29

**Authors:** Shyam Gopalakrishnan, Jose A. Samaniego Castruita, Mikkel-Holger S. Sinding, Lukas F. K. Kuderna, Jannikke Räikkönen, Bent Petersen, Thomas Sicheritz-Ponten, Greger Larson, Ludovic Orlando, Tomas Marques-Bonet, Anders J. Hansen, Love Dalén, M. Thomas P. Gilbert

**Affiliations:** 1 Centre for GeoGenetics, Natural History Museum of Denmark, University of Copenhagen, Øster Voldgade 5-7, 1350 Copenhagen, Denmark; 2 Natural History Museum, University of Oslo, N-0318 Oslo, Norway; 3 Institute of Evolutionary Biology (UPF-CSIC), PRBB, Dr. Aiguader 88, 08003 Barcelona, Spain; 4 CNAG-CRG, Centre for Genomic Regulation (CRG), Barcelona Institute of Science and Technology (BIST), Baldiri i Reixac 4, 08028 Barcelona, Spain; 5 Department of Environmental Research and Monitoring, Swedish Museum of Natural History, Box 50007, 10405 Stockholm, Sweden; 6 Department of Bio and Health Informatics, Technical University of Denmark, 2800 Kongens Lyngby, Denmark; 7 Palaeogenomics & Bio-Archaeology Research Network, Research Laboratory for Archaeology and the History of Art, University of Oxford, OX1 3QY, Oxford, UK; 8 Catalan Institution of Research and Advanced Studies (ICREA), Passeig de Lluís Companys, 23, 08010 Barcelona, Spain; 9 Department of Bioinformatics and Genetics, Swedish Museum of Natural History, Box 50007, 10405 Stockholm, Sweden; 10 Trace and Environmental DNA Laboratory, Department of Environment and Agriculture, Curtin University, Perth, Western Australia, Australia; 11 NTNU University Museum, Norwegian University of Science and Technology, Trondheim, Norway

**Keywords:** *Canis lupus*, Evolutionary genomics, Genome, Wolf

## Abstract

**Background:**

An increasing number of studies are addressing the evolutionary genomics of dog domestication, principally through resequencing dog, wolf and related canid genomes. There is, however, only one de novo assembled canid genome currently available against which to map such data - that of a boxer dog (*Canis lupus familiaris*). We generated the first de novo wolf genome (*Canis lupus lupus*) as an additional choice of reference, and explored what implications may arise when previously published dog and wolf resequencing data are remapped to this reference.

**Results:**

Reassuringly, we find that regardless of the reference genome choice, most evolutionary genomic analyses yield qualitatively similar results, including those exploring the structure between the wolves and dogs using admixture and principal component analysis. However, we do observe differences in the genomic coverage of re-mapped samples, the number of variants discovered, and heterozygosity estimates of the samples.

**Conclusion:**

In conclusion, the choice of reference is dictated by the aims of the study being undertaken; if the study focuses on the differences between the different dog breeds or the fine structure among dogs, then using the boxer reference genome is appropriate, but if the aim of the study is to look at the variation within wolves and their relationships to dogs, then there are clear benefits to using the de novo assembled wolf reference genome.

**Electronic supplementary material:**

The online version of this article (doi:10.1186/s12864-017-3883-3) contains supplementary material, which is available to authorized users.

## Background

In light of the ever-decreasing cost of high-throughput DNA sequencing, it is now possible to undertake large-scale genomic studies at not only the population level, e.g. [1, 2], but also the population paleogenomic level, e.g. [3–8]. While these datasets are being exploited across a growing range of applied questions, a number of research groups are beginning to also focus on how to interpret and treat this data in a way that minimizes biases, and thus yields robust inferences from the data.

Several human population genomic datasets have noted the existence of biases that arise when mapping the resequenced genomes of diverse individuals to a reference genome based on a single individual. Alignment against a single reference genome can lead to different samples appearing more similar to the reference genome, and underestimating the variation present in samples that come from a different population or species than the reference genome [9–11]. New mapping techniques are being developed to overcome these biases by allowing mapping to multiple genomes [12]. These methods rely on a high number of sequenced and de novo assembled samples, or a catalogue of polymorphisms for all the populations in the study. For species other than humans, such resources are scarce. Ultimately, these biases imply that thorough annotation of all variation in a genomics data set requires every individual to be represented by a de novo assembly [13–15]. Though this ideal is not feasible for a variety of economic reasons, there is a need to broaden the pool of reference genomes to ensure that we can minimize the effects of these biases on downstream analyses.

A research discipline where population genomics is rapidly making significant contributions is the study of domestication – a topic that has long held academic interest due to both its applied relevance and its broad general public appeal. Genomic and paleogenomic resources have previously been used to address major questions in domestication, including deciphering the population structure and admixture patterns in modern and wild lineages [16–18], discovering strcuture among ancient pre-domestic lineages [6, 19–22], and estimating levels of introgression from wild lineages into domesticated stocks [17, 23], applied to a multitude of species, such as maize [6, 16, 22], silkworms [24], chickens [25–27], and pig [28, 29].

Although these analyses can offer powerful insights into the domestication process, they come with their own sets of challenges. While the major challenge is the need to account for genetic diversity that has been lost as a result of full or partial extinctions of original wild lineages, mapping biases arising from experimental design, such as choice of reference genome, also pose a hurdle to robust analyses. At least one domestication related study has demonstrated that these effects can be considerable. In Orlando and colleagues’ [19] study of the genomic sequences of six horses (one from a pre-domestication Pleistocene sample), they showed how a variety of analyses such as D statistics, population divergence and heterozygosity estimation, led to different results when their resequenced genomes were mapped to the EquCab2.0 [30] reference genome, and a de novo assembly of the donkey genome. They attribute many of these biases to differences in how closely related the samples are to the horse reference genome. This problem is exacerbated in studies that include ancient, pre-domestication samples since the reference genomes are predominantly constructed using modern samples. Another difference in the reference genomes that might lead to different results in downstream analyses, is the technology used to generate the reference genome. Many older reference genomes were generated using Sanger sequencing while the newer reference genomes and resequenced genomes in studies have been generated using Illumina short read sequencing technology. Although the underlying causes for the biases remain unresolved, one powerful approach is to perform the analyses using several different closely related reference genomes, thus accounting for biases introduced by the mapping procedures and ensuring that the results are consistent across the choice of reference genomes.

With regards to the need for multiple reference genomes, while a number of genomics studies have recently been published that relate to the relationship between dogs and wolves, the sequence data from genome resequencing studies [21, 31–34] has either been mapped to the only currently available reference genome, that of the Boxer dog (CanFam3.1) [35], or compared to data drawn from SNP (Single Nucleotide Polymorphism) chip arrays developed to target variation in dog genomes [36, 37]. The results of such studies show that dogs are monophyletic with respect to wolves, and indicate the existence of a deep split between the modern wolf and dog lineages, and a deep split within the dogs as well [21].

There are still several questions regarding wolf and dog phylogeny, population history and domestication that remain unanswered. Although the results of these studies are largely consistent, there are some inconsistencies in the findings regarding the location and the time of the domestication event [21, 36, 38, 39]. It has also been suggested that the population of wolves that are ancestral to the modern dogs may be extinct [21, 32, 34].

It is possible that one explanation for discrepancies between studies is that important structural variation in the wolf genome is missed or misplaced by mapping to a dog reference, or targeting SNPs developed for dog variation. To test this hypothesis, we de novo generated the first wolf reference genome, then remapped the genomic datasets previously published by Wang et al., Freedman et al. and Zhang et al. [31–33]. We subsequently re-analysed the published and remapped data in the context of divergence, admixture and systematics, in order to explore whether any reference genome-specific biases occur.

## Results

### De novo reference genome assembly

In order to construct a de novo reference genome using a wolf, we generated a combination of 5–8 kilobases and 3 kilobases mate pair libraries, as well as 650 basepair and 180 basepair insert libraries. These were sequenced with 101 basepair paired end reads using 5 lanes of a Illumina Hiseq 2500, where one lane was allocated to the multiplexed mate-pair libraries, one lane to the 650 basepair insert library and the remaining three lanes were allocated for the 180 basepair insert libraries. Overall, this generated a 30× coverage of the genome. The de novo reference genome was assembled using the ALLPATHS-LG assembler [40]. The final assembly consisted of 8747 scaffolds, of which 8569 scaffolds were longer than 1 kilobase. The longest scaffold was 12.88 megabases. The scaffold N50 of the assembly is 1.56 megabases and the scaffold N80 of the assembly was 512 kilobases., the contig N50 of the assembly was 94 kilobases and the contig N80 of the assembly was 34 kilobases. The total length of the assembly was 2.34 gigabases, while the scaffolds longer than 1 kilobase covered more than 99.99% of the assembly.

### Landscape of common repeats

To compare abundances of repetitive elements between the wolf assembly and canFam3, we sought to detect common interspersed repeats in both of them. We identify 902 megabases of repetitive elements along the wolf assembly, correspoding to 39.8% of the non-gapped assembly. We detect a similar, albeit slightly higher amount of repeats in canFam3 (1009 megabases, or 42.1% of the non-gapped assembly). When stratifying repetitive elements by their respective superfamilies, we observe simliar abundancies in the wolf and the dog assembly (see Additional file 1: Figure S2), with the exception of satellite sequences, a family of repetitive elements most commonly found in the telomeric and centromic regions of the chromosomes. To investigate the patterns underlying the differences in repeat annotations, we calculated the evolutionary distance of each annotation to its consesus sequence. Overall, the divergence landscapes are very similar, however, we observe a depletion of young and highly identical long interspersed nuclear elements (LINEs) and short interspersed nuclear elements (SINEs) insertions in the wolf assembly, most likely as an artifact of sequencing and assembly strategy (see Additional file 1: Figure S3).

### Mapping, coverage statistics

Since the choice of reference genome directly affects the mapping process, we compared the efficiency of mapping previously published short reads to the reference genome when using one of the two genomes used in this study, viz., the dog reference genome [35] and the de novo assembled wolf reference genome. We compared the proportion of uniquely mapped reads for each sample and the depth of coverage across the genome. As shown in Table S1 (Additional file 1: Table S1), we find that the samples that come from the same sub-species as the reference genome, i.e. dogs when using the dog reference genome and wolves when using the wolf reference genome, have a higher proportion of reads that map uniquely to the genome. As a result, they also have a slightly higher coverage across the genome. Note that we do not find a large difference in coverages or proportions of reads that map uniquely, and the effect is consistent across all samples.

### PCA

**Fig. 1:**
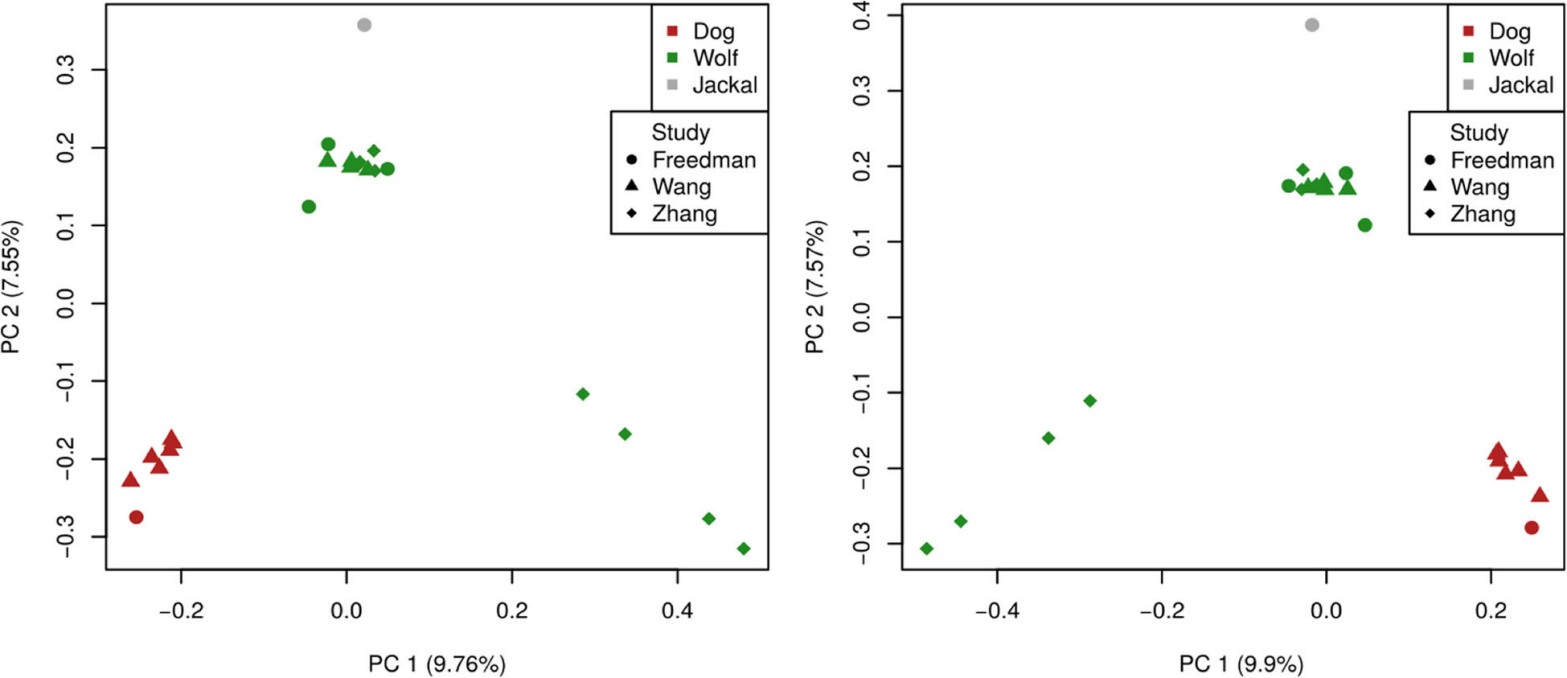
Principal components analysis comparing the first two PCs when using the wolf or the dog reference genome. The left panel shows the principal components analysis when using the variants obtained from mapping to the dog genome, and the right panel shows the same when using variants obtained from mapping to the wolf genome

We performed a principal components analysis (PCA) to identify the major axes of variation in the genotype data. Fig. 1 shows the results of the PCA using data mapped to either the reference dog or the de novo wolf genome assembly. For this analysis, we used only common variants with minor allele frequency greater than 0.05. Irrespective of the reference genome used for the aligment, the first two principal components separate dogs from wolves. The proportions of variance explained by the first and second principal components are also very similar across the choice of the two reference genomes (see Fig. 1). Changing the missingness or allele frequency threshold leads to qualitatively similar results (Additional file 1: Figure S4).

### Heterozygosity

We compared the estimates of per-sample heterozygosity using alignments to the two different reference genomes. Table S1 (Additional file 1: Table S1) shows that the estimated heterozygosity of the samples depends upon the reference genomes used for mapping. The heterozygosity estimates for dogs are consistently higher by upto 10% when using the dog reference genome compared to the de novo wolf genome assembly.

### Population size

**Fig. 2:**
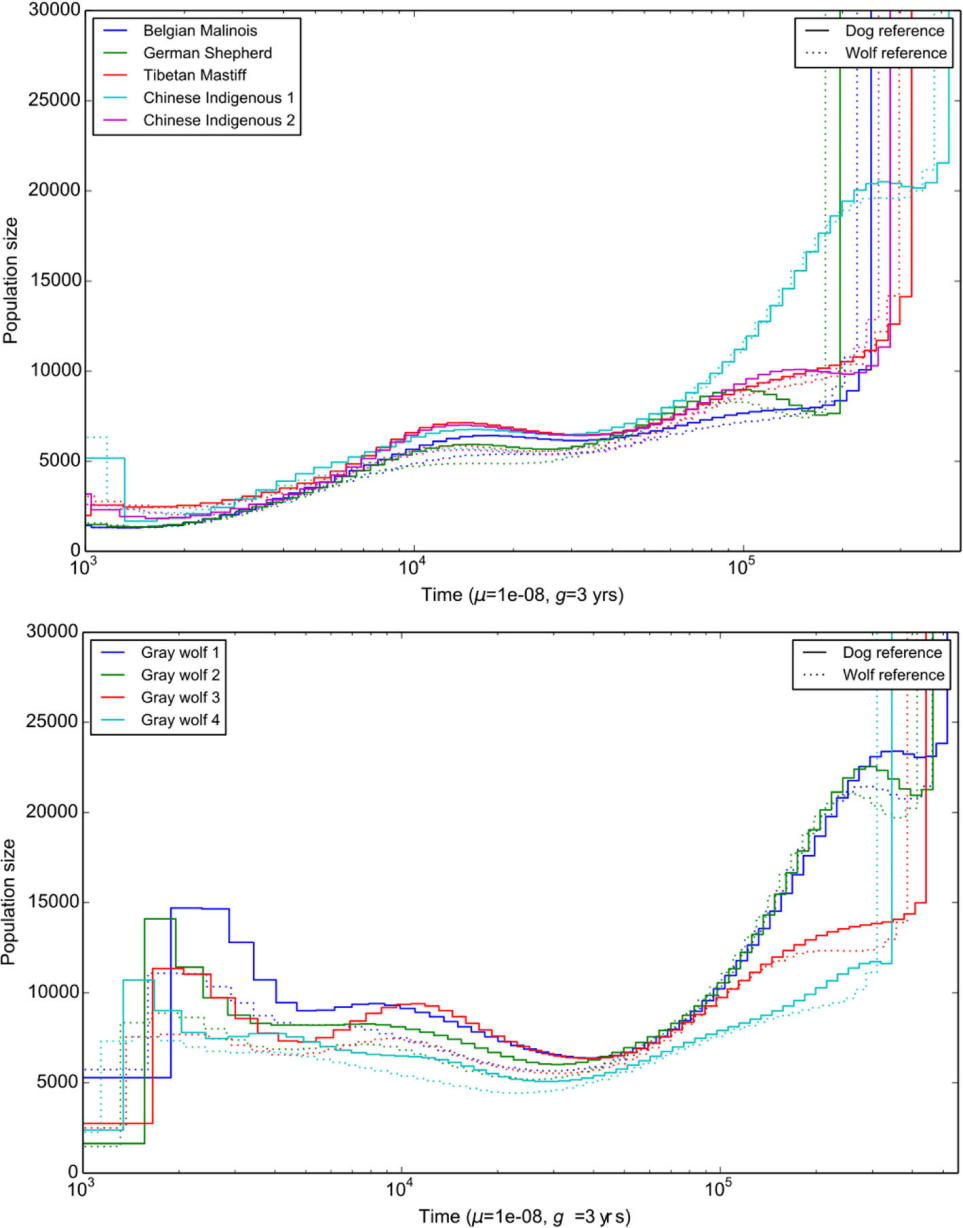
Effective population sizes estimated using PSMC. The left panel shows the effective population sizes for dogs in the Wang dataset, estimated using the data mapped to both the reference genomes. The right panel shows the population size estimates for the wolves in the Wang dataset, when using the data mapped to both the reference genomes

We additionally used the pairwise sequentially markovian coalescent (PSMC) [41] to explore the effect of reference genome on the estimated population size history of the populations that the resequenced individuals were obtained from. Figure 2 shows the reconstructed population size history for a subset of the samples in our study. The comparison of the population sizes shows that the estimates obtained are largely consistent. For the dogs in this study, the population size trajectories estimated using the two different reference genomes coincide beyond 10kya. However, the effective population sizes for the wolves are a bit lower when using the wolf reference genome, compared to the same when using the dog reference genome. We observed reference genome specific differences in the recent histories, which can be attributed to the difference in the rare/private variants discovered in the two species when using the different reference genomes. If the primary effect of changing the reference genome is in the number of rare variants discovered, the effect on analyses such as PSMC will be greatest in the recent population size estimates. As PSMC does not have the power to estimate these parameters well, the effect of this bias is not expected to be high in this analysis.

### Phylogeny

We used *RAxML* and *ExaML* [42, 43] to estimate the phylogenetic relationships between samples using the variants identified by aligning to the wolf or the dog reference genome. Since our analysis only uses variant sites, we accounted for the ascertainment scheme of the variants using the ascertained version of the GTRGAMMA model of sequence evolution. As shown in Additional file 1: Figure S1, the overall topology of the resulting phylogenies differ depending on the choice of the reference genome. Specifically, when using the dog reference genome the dogs and wolves are reciprocally monophyletic. While using the de novo assembled wolf reference genome, the dogs were monophyletic with respect to the wolves but the wolves were not monophyletic with respect to the dogs. Note that the support values for these nodes that differ between the two topologies have very low bootstrap support values. Additionally, using a neighbour joining approach to estimate the phylogenetic relationships led to qualitatively similar results (data not shown).

### Admixture

**Fig. 3:**
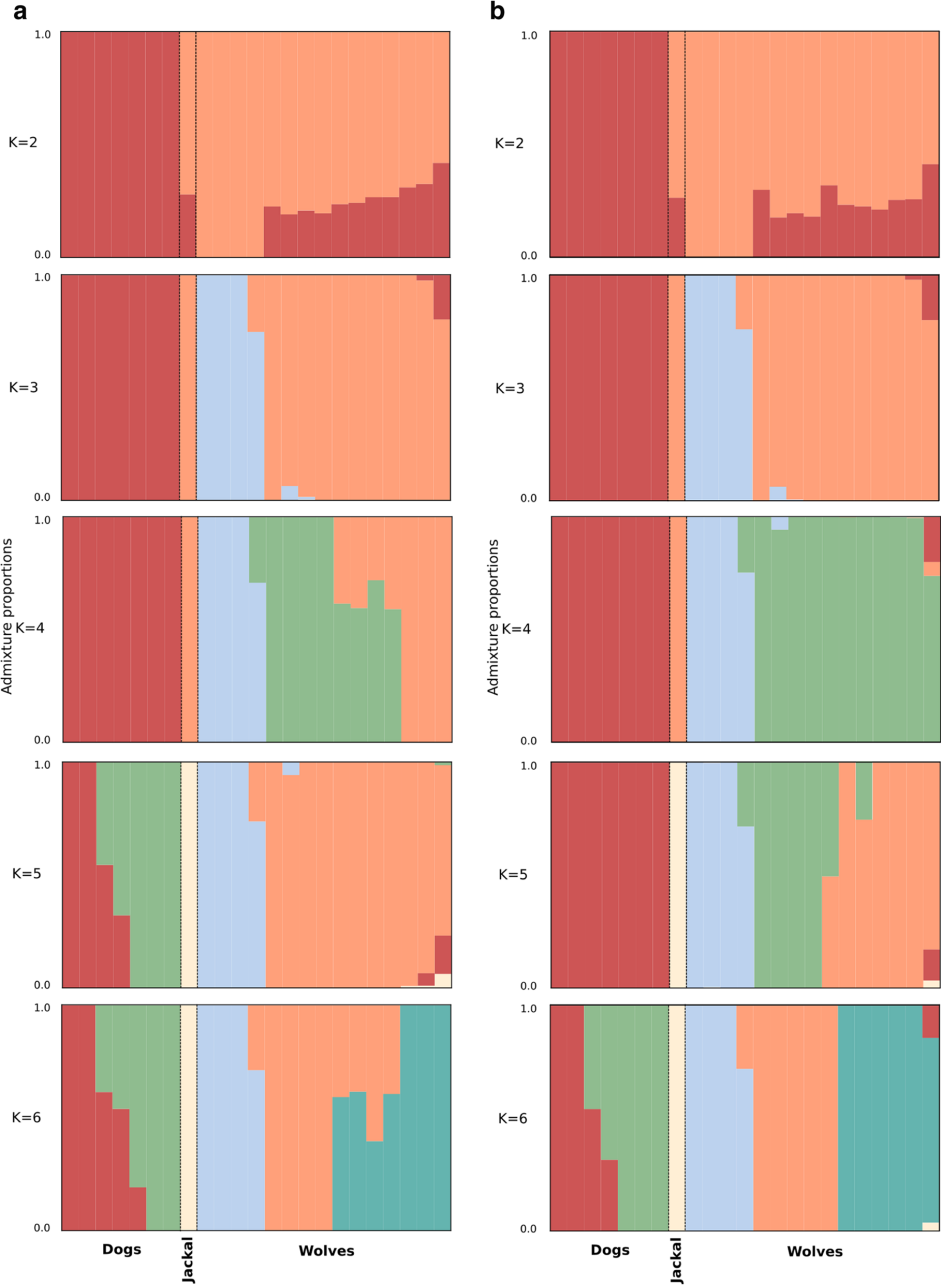
Admixture plot showing the estimated ancestry components. The plots on the left, panel **a**, show the estimates obtained when the dog reference genome is used, whereas the figures on the right, panel **b**, were obtained using the wolf reference genome

We estimated the ancestry proportions in the 23 samples using ngsAdmix [44]. When using two ancestry components for estimating admixture proportions, dogs and wolves are split into two different clusters for both choices of reference genome. In both cases, all the wolves, except for the high altitude wolves from the Zhang study [33], show up to 20% of the estimated dog ancestral component (Fig. 3). Increasing the number of estimated ancestral components from two to three leads to similar results, with the dogs and the wolves being separated into two clusters. Additionally, the wolves split into two clusters where the high altitude wolves are separated from the rest of the wolves. Further, the contribution of the estimated dog ancestry components in the wolves becomes negligible.

When estimating admixture with four ancestry clusters, the choice of the reference genome has an impact on the qualitative outcome of the admixture analyses. When using the de novo wolf reference genome, the newly added ancestry component separates the golden jackal (*Canis aureus*) from the other samples, whereas using the boxer dog reference genome reveals additional structure in the wolves, with the golden jackal assigned to one of the clusters containing the wolves. When estimating a higher number of ancestry components, the additional ancestry components explain variance in dogs if the dog reference genome was used and conversely, the use of the de novo wolf reference genome leads to additional structure in the wolves.

## Discussion

Previous studies have speculated that the choice of reference genome has wide ranging effects, especially on the identification of population structure and the timing of demographic events in studies using multiple related species. This problem is expected to be exacerbated when the reference genome is closer to some species in the study than others. Given that there is currently a considerable amount of effort being applied to the sequencing and analysis of dog and wolf genomes, we decided to both explore the impact of the phenomenon in general, and specifically explore whether it holds implications for the results of several relevant previously published dog and wolf genome studies. In this regard, because the time of divergence between dogs and wolves is relatively recent (a conservative estimate of the divergence time is around 35,000 years ago [31, 34]) and the genetic divergence between the extant wolves and modern dogs is low, we did not, a priori, expect the choice of the reference genome to have a big impact on the qualitative inferences in the standard population genetics analyses. Overall, our findings bear this expectation out - the analyses that are primarily driven by common variation, such as principal components analysis and admixture analysis with low number of clusters result in very similar findings across the two reference genomes.

Nevertheless, since these two species *are* genetically very similar, the rare and/or private variation is informative for the differences between the two species. Regarding these variants, the choice of reference genome is clearly more important than for the common variants. As shown in both the table of heterozygosity (Additional file 1: Table S1) and the results from admixture analyses with higher number of estimated ancestry clusters (Fig. 3), the rare variation in the two datasets can lead to qualitatively different results. This is especially evident in the admixture analyses with four or more clusters, where the structure that is revealed is dependent on the choice of the reference genome. Using the data aligned to the dog genome results in earlier identification of structure in dogs, and vice versa.

One main concern when interpreting these results is the differences in the quality of the two reference assemblies. Clearly, the dog reference genome is in a much more mature state than our de novo assembly of the wolf reference genome. This difference in quality could lead to biases in the analyses, especially analyses that require large continuous regions with variant calls, e.g., effective population size estimation using PSMC as well as characterization of inbreeding levels using runs of homozygosity. Although the effective population size estimates are consistent for the two reference genomes, the difference in quality of assembly could result in different estimates in the most recent time periods, where the methods are typically underpowered.

The effect of the choice of the reference genome seems to be limited to analyses that rely of low frequency and private variants. When comparing the effect from mapping against wolf and dog reference genomes, we found the largest effect in the higher order structure identified in the wolves or dogs when estimating ancestry components. At lower number of ancestry components, the choice of reference genome had no effect on the identification of clusters.

In this study, neither of the two reference genomes used were equally distant from the wolves and dogs samples analysed. Ideally, one could use the genome of a relatively close outgroup – the golden jackal in our case – to ensure that there are no biases introduced due to the choice of the reference genome. Although this would avoid the pitfalls of choosing a reference genome that is more close to some of the samples than others, it may not be feasible in many cases, e.g. due to the relatively high economic and computational costs of generating outgroup genomes, or the absence of an appropriate outgroup. Since the reference genomes for most studies tend to not be equally distant from all samples, it is important to account for the biases while interpreting the findings from population and phylogenetics analyses.

## Conclusions

We have generated the first de novo assembled wolf reference genome, which will be a useful resource for future studies exploring the genomic structure and relationship between dogs, wolves and other canids. Since the two species that are the focus of this paper are so closely related, the effect of the reference genome was minimal on many of the downstream analyses such as PCA and estimating the phylogeny of the samples. However, some analyses like admixture showed the effects of the reference genome at higher number of clusters. Since the use of the wolf reference genome results in identification of population structure that is hidden when using the dog reference genome, we recommend the use of the de novo wolf reference genome for any studies where the focus is on identifying the relationships between wolves and dogs or teasing apart the relationship between the various wolves of the world.

## Methods

### De novo reference genome assembly

We used a muscle sample from a Swedish wolf to construct (Additional file 1: Figure S5) our de novo reference genome. The sample (specimen ID: NRM201105024) was obtained from the Environmental Specimen Bank at the Swedish Museum of Natural History, and originated from a male yearling shot during a licensed hunt in Värmland, Sweden in January 2011. The individual (Grimsö ID: D-11-21) was born in the Jangen 5 territory [45]. The pedigree-based inbreeding level (F) for the offspring born in this territory has been estimated to be F = 0.30 [46].

In order to generate a de novo wolf reference genome assembly, we created libraries with different insert sizes, viz., one 5-8 kb mate pair library, one 3 kb mate pair library, and 650 bp and 180 bp insert libraries. In all, these libraries were sequenced using 5 lanes of Hiseq 2500. Using the short reads generated from these libraries, the de novo reference genome was assembled at NGI Stockholm using the ALLPATHS-LG [40] assembler. Different assemblers were tested before choosing the genome assembled using the ALLPATHS-LG assembler, based on statistics of the assembly, such as the number of scaffolds, the N50, N80 and total assembly length.

### Repeat identification

To identify common genomic interspersed repeats in the wolf assembly, we ran RepeatMasker [47] (version 4.0.6) with RMBLAST (version 2.2.27+) as engine. We used the dog-specific repeat libraries derived from the latest available Repbase database (version 20,160,829, available at www.girinst.org). To put our results into context, we also identified interspersed repeats with the exact same approach in the latest dog reference genome assembly canFam3 [35], as published annotations might differ slightly in parameter settings or engine.

### Resequencing data used in comparisons

To quantify the effects of the choice of the reference genome on the downstream analyses, we used publicly available datasets that contained whole genome sequences for canids. We used the raw short reads from the sequenced wolves and dogs from Freedman et al., Wang er al. and Zhang et al. [31–33]. From Freedman et al., we obtained the short reads for the 6 canids that were whole-genome sequenced as part of their study, viz., a dingo, an Israeli wolf, a Croatian wolf, a Chinese wolf, a basenji and a golden jackal. Of these samples, we did not use the basenji due to data corruption issues. From the Wang et al. study, we downloaded the short reads for four gray wolves, three Chinese indigenous dogs, two European dog breeds – the German shepherd and the Belgian Malinois – and a Tibetan mastiff. We also obtained the shotgun short reads from the whole genomes of 8 Chinese wolves that were sequenced as part of the Zhang et al. study.

The details of the samples included in our study, including the sequencing depth of coverage, and the source from which the data were obtained are given in Table S1 (Additional file 1: Table S1).

### Data processing

Since the different datasets that were used in this study were obtained from various different sources, we built a custom processing pipeline to ensure that all the data were processed using the same tools and were subject to the same quality control and filtering. We built our pipeline on the Paleomix pipeline developed by Schubert et al. [48]. The various parts of our processing pipeline are detailed below.

### Mapping

Each sample used in this study was mapped against both the dog reference genome (canFam3.1) [35] and the de-novo assembled wolf reference genome. We used the Paleomix pipeline to map the short reads from the samples to both the genomes. Specifically, we used bwa-0.7.10 (the aln algorithm) [49] to map the reads to the genome. After the initial alignment step, we discarded any reads that did not map uniquely to the reference genome. Using only the uniquely mapped reads, we used GATK [50] to perform an indel realignment step to account for increased error rates in reads whose ends span an indel. As there are no available curated sets of indels for the dog or the wolf populations, we did not use an external database of indels for indel realignment.

Since the aim of this paper is to compare the choice of reference genome, mapping to the reference genome is a critical step in the bioinformatics processing. Any biases or errors introduces as part of the mapping process will propagate to the downstream analysis resulting in incorrect inferences. In order to ensure that we did not introduce any such biases, we used exactly the same settings while mapping to the wolf or the dog reference genomes.

### Genotyping

After mapping the reads to the reference genome, we called genotypes for all sites in the genome. Since the samples in this study consist of different populations and species, each sample was processed independently. For each sample, the positions in the genome that were covered by at least 5 reads were genotyped. At each such site, the genotype was called using *samtools-0.1.19* [49, 51]. We used a minimum genotype quality threshold of 30 to filter out low quality gentotypes from each sample.

### Variant identification and quality filtering

Using the initial set of genotypes for all samples at all sequenced sites, we identified variant sites in the study sample. We did not use a multi-sample variant called since we have a heterogenous set of samples. Since we did not use a multi-sample variant caller, the variants identified will consist of a lot of false positives. We used multiple different filters to exclude false positives and get a final set of analysis ready variants. Similar to the Freedman et al. study, we used different sets of filters for different analyses. The filtering criteria are detailed below.

#### Genotype and variant quality

We marked all genotypes that had a phred scaled genotype quality of less than 30 as missing genotypes. Further, we also excluded variants that had a variant quality of less than 20.

#### Depth of coverage

For each sample, we excluded sites that had an abnormally low or high coverage compared to the rest of the genome. We removed any sites that did not have at least 5 reads covering that site, since the uncertainty in the genotype call is high when it is based on a low number of reads. In addition, we also excluded any sites that had more than twice the average genome-wide coverage. The rationale behind discarding sites with high coverage is that these sites have a high coverage either due to mapping artifacts or the presence of homologs in the genome.

#### Distance to neighboring variants

The presence of indels can cause the identification of false positive variants due to mapping artefacts around the indel. Similarly, a cluster of SNPs close to each other is an indicator of mapping artifacts. To filter out these false variants arising from mapping artifacts, we filter out variants that within 5 base pairs of another SNP or indel. Further, we used a lower quality score threshold for identification of neighboring variants, i.e., variants with a quality score between 10 and 20 were included in the pool of variants when filtering for distance to other variants. This ensures that we filter out any SNPs that are close to other variants, even if the neighboring variants do not pass our quality filters.

#### Triallelic single nucleotide polymorphisms (SNPs)

We identified triallelic SNPs across the 23 samples. We used a genotype quality threshold of 30 and a variant quality threshold of 20 to identify such variants. We excluded all tri-allelic sites from downstream analyses.

#### Minor allele frequency

We used multiple different minor allele frequency (maf) thresholds to prune our data depending on the analysis. For all the analyses performed in this study, we excluded singletons i.e. variants with only one copy of the rare allele in the sample. For PCA and admixture analyses, we used two different maf thresholds (0.05–0.2) to obtain different datasets on which we repeated the analysis to explore the effect of low frequency variants on the analysis.

#### Missingness

We excluded variant sites that had a high proportion of samples with missing genotype calls. These sites were excluded for analysis that required called genotypes. Missingness threshold dependent upon the analysis that are performed. For each of these analysis, the missingness threshold is indicated in the relevant section.

### Principal components analysis

As a first step to assessing the effect of reference genomes on the outcome of the data, we performed a principal components analysis (PCA) on the 23 samples. From the genotypes obtained for the samples after aligning to the dog reference genome, we identified variants in the combined set of samples. We excluded any variants which had a minor allele frequency less than 0.05. We also discarded variant sites with greater than 5% missingness. We used three additional filters to prune our dataset: triallelic SNPs, distance to nearest variant and depth of coverage. Using this filtered dataset, we performed PCA using the ngsCovar program available as part of the ngsTools suite of tools [52]. We repeated the analysis with different levels of missingness (10%, 20%) and minor allele frequency thresholds (0.05, 0.1 and 0.2) to check the robustness of our findings. We performed an identical analysis using the alignments obtained from mapping to the de novo wolf genome.

### Heterozygosity

To compute the heterozygosity per sample, we used the per sample genotype calls and excluded sites with a genotype quality less than 20 and a variant quality less than 30. We also discarded sites that were within 5 basepairs of another variant (indel or SNP) with a variant quality greater than 10. Using this filtered set of variants, we used *plink* [53] to compute the heterozygosity for each sample.

### Population size (PSMC)

We used pairwise sequentially markovian coalescent (PSMC) [41] approach to compute the population size history of the samples in our dataset. For each sample, we used the genotypes obtained from the alignments to the dog or the de novo wolf reference genome, to obtain a consensus fasta sequence across the entire genome. We filtered out sites with genotype quality lower than 20 or site quality lower than 30. Further, we used the depth filter to exclude sites with abnormally low or high number of reads covering it. Finally, we excluded variant sites that were less than 5 basepairs away from another variant site with quality score greater than 10. We generated the PSMC input file using a window size of 100 base pairs. During this process, we marked all windows with more than 80 unknown/missing bases as missing. Using this input file, we ran psmc by dividing time into 64 bins, and used the pattern of “1*6 + 58*1” to estimate 59 independent population size parameters.

### Phylogenetic analysis

We constructed the phylogeny of all the samples using the variants identified by mapping the reads either to the de novo wolf reference assembly or the publicly available dog reference genome. For this analysis, we filtered out variants that were closer than 5 bp from another variant. In addition, we also excluded variants with quality scores lower than 30 and genotypes with quality scores less than 20.

We used the paleomix pipeline to obtain the phylogeny from these variants. As part of the *paleomix* pipeline, we used *RAxML* and *ExaML* [42, 43] to estimate the phylogeny of these samples. Since we do not have the annotations of genes for the de novo wolf reference wolf genome, we used 5 megabases of sequences - 100 randomly selected regions, each 5 kb long – to estimate the phylogeny. We generated the consensus sequences for each of the samples using *samtools-0.1.19* [49, 51]. Since all the samples are mapped against the same genome and the indels are discarded, the multiple alignment of these regions were readily obtained by matching the genomic positions of the regions across samples. Using RAxML and ExaML, we generated the phylogeny of all the samples. We used 5 random starting points to generate 5 replicates from the data. We used 100 bootstrap runs to obtain the support for the nodes in the tree.

### Admixture

We performed an admixture analysis to identify structure and admixture in the samples. From the variants identified by combining the genotypes from all the samples, we excluded sites with qualities lower than 30, missingness greater than 25% and minor allele frequencies less than 5%. In addition, we also marked all genotypes with qualities lower than 20 as missing.

We used ngsAdmix [44] on this filtered dataset to obtain the admixture proportions in the samples. We ran ngsAdmix for different numbers of clusters, ranging from 2 to 6. For each value of the number of clusters, we ran the analysis 10 times and chose the run that gave us the best likelihood at convergence. Similar to our other analyses, we performed the same analysis for data obtained from mapping to the dog reference genome and the data from mapping to the de novo wolf reference genome.

## Additional file

Additional file 1: Figure S1. Phylogenies. The left panel shows the phylogenetic tree of all the samples, estimated from reads that are mapped to the boxer dog reference genome, while the right panel shows the phylogenetic tree estimated from the data after mapping reads to the denovo assembled wolf reference genome. **Figure S2.** Distribution of repeat elements. Total amount of bases in different repeat classes across the two reference genomes. **Figure S3.** Comparison of the divergence of the different repeat elements from their consensus sequence. The top panel shows the total number of bases against the divergence from the consensus sequence in each repeat family when using the de novo wolf reference genome for alignment. The bottom panel shows the same figures when using the boxer reference genome. **Figure S4.** Principal Components Analysis (PCA). Panels A and B show the first four principal components of the genotypes when using the de novo wolf reference assembly. For making these PCA plots, we used a missingness cutoff of 0.9 and a minor allele frequency cutoff of 0.2. Panels C and D show the first four principal components of the genotypes when using the boxer reference genome while using the same filtering thresholds. **Figure S5.** Picture of the skull of the Swedish wolf sample used for reference genome assembly. **Table S1.** Coverage and heterozygosity estimates. The coverage and heterozygosity are shown for each sample included in the study. For each animal, the higher estimate of coverage are bolded. (PDF 1652 kb)

## Abbreviations

LINE: Long interspersed nuclear elements
PCA: Principal components analysis
PSMC: Pariwise sequentially markovian coalescent
SINE: Short interspersed nuclear elements
SNP: Single nucleotide polymorphism
