# Supplementary material for: The wolf reference genome sequence (Canis lupus lupus) and its implications for Canis spp. population genomics
Source: BMC Genomics. 2017 Jun 29;18:495. doi: 10.1186/s12864-017-3883-3 (PMC5492679; doi:10.1186/s12864-017-3883-3)
Supplement: Additional file 1: Figure S1. — Phylogenies. The left panel shows the phylogenetic tree of all the samples, estimated from reads that are mapped to the boxer dog reference genome, while the right panel shows the phylogenetic tree estimated from the data after mapping reads to the denovo assembled wolf reference genome. Figure S2. Distribution of repeat elements. Total amount of bases in different repeat classes across the two reference genomes. Figure S3. Comparison of the divergence of the different repeat elements from their consensus sequence. The top panel shows the total number of bases against the divergence from the consensus sequence in each repeat family when using the de novo wolf reference genome for alignment. The bottom panel shows the same figures when using the boxer reference genome. Figure S4. Principal Components Analysis (PCA). Panels A and B show the first four principal components of the genotypes when using the de novo wolf reference assembly. For making these PCA plots, we used a missingness cutoff of 0.9 and a minor allele frequency cutoff of 0.2. Panels C and D show the first four principal components of the genotypes when using the boxer reference genome while using the same filtering thresholds. Figure S5. Picture of the skull of the Swedish wolf sample used for reference genome assembly. Table S1. Coverage and heterozygosity estimates. The coverage and heterozygosity are shown for each sample included in the study. For each animal, the higher estimate of coverage are bolded. (PDF 1652 kb) [file 12864_2017_3883_MOESM1_ESM.pdf]

SUPPLEMENTARY MATERIAL

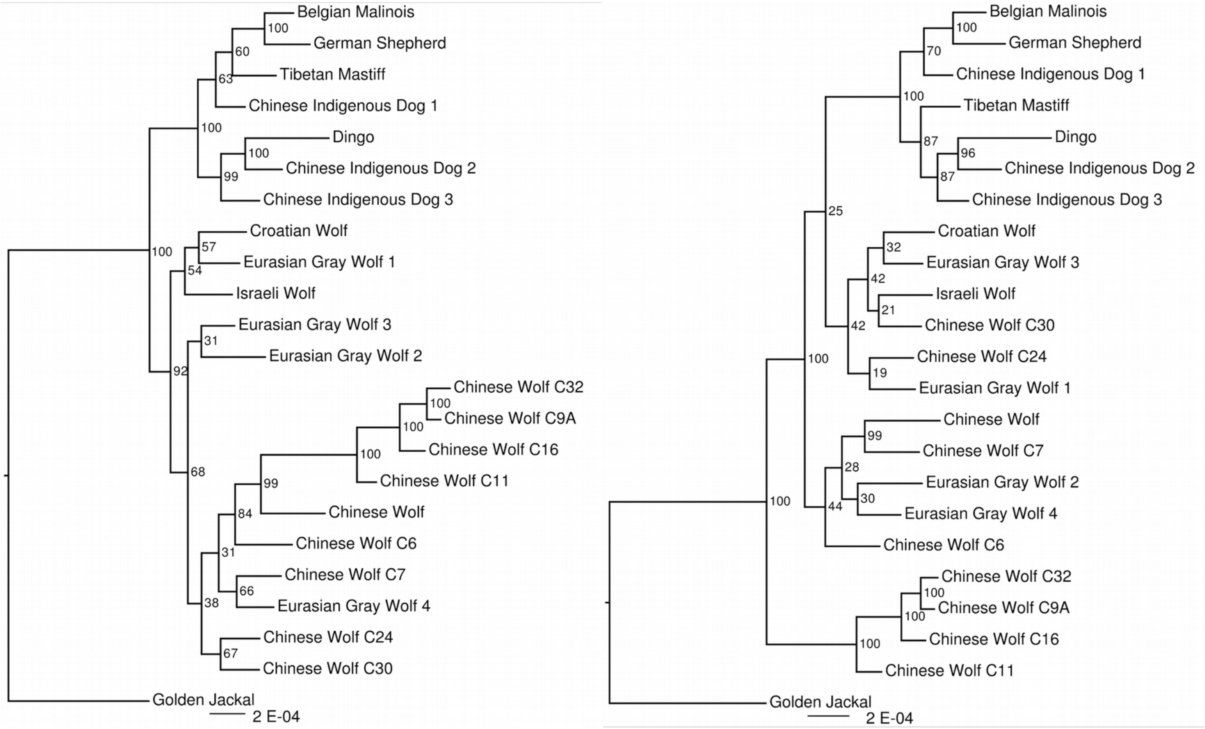

Supplementary Figure 1: Phylogenies. The left panel shows the phylogenetic tree of all the samples, estimated from reads that are mapped to the boxer dog reference genome, while the right panel shows the phylogenetic tree estimated from the data after mapping reads to the de novo assembled wolf reference genome.

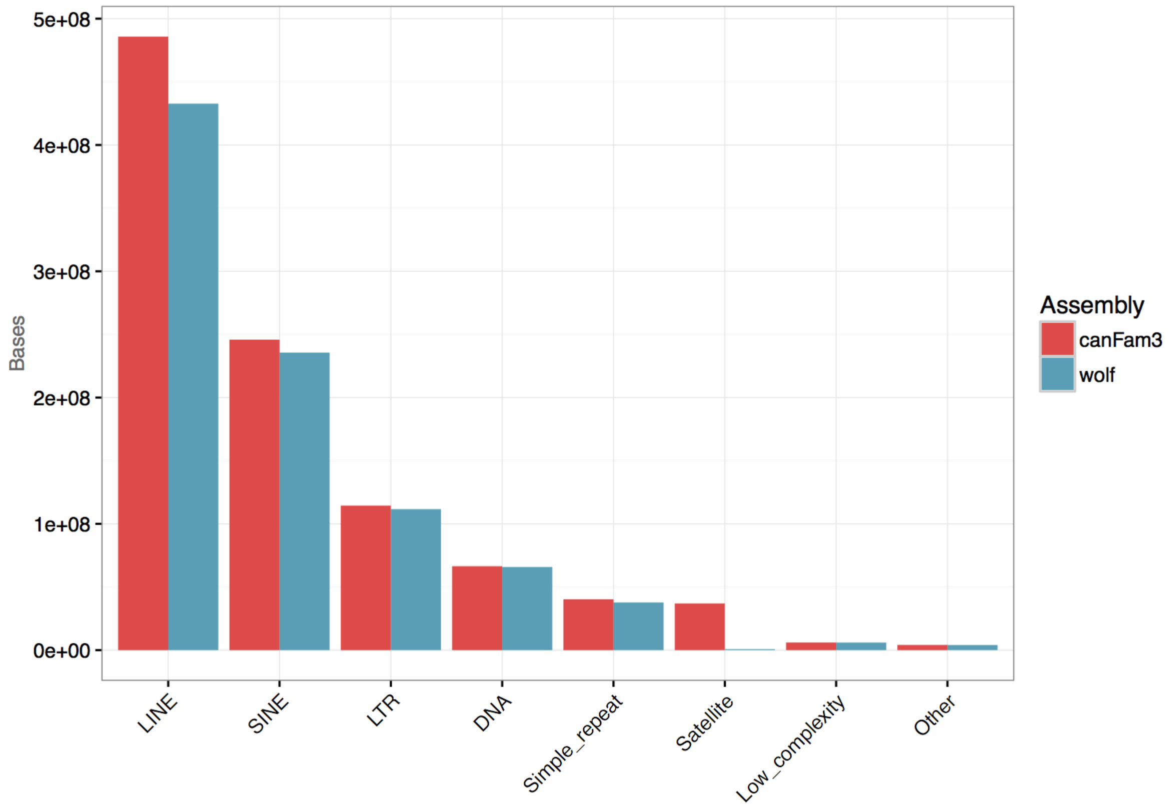

Supplementary Figure 2: Distribution of repeat elements. Total amount of bases in different repeat classes across the two reference genomes.

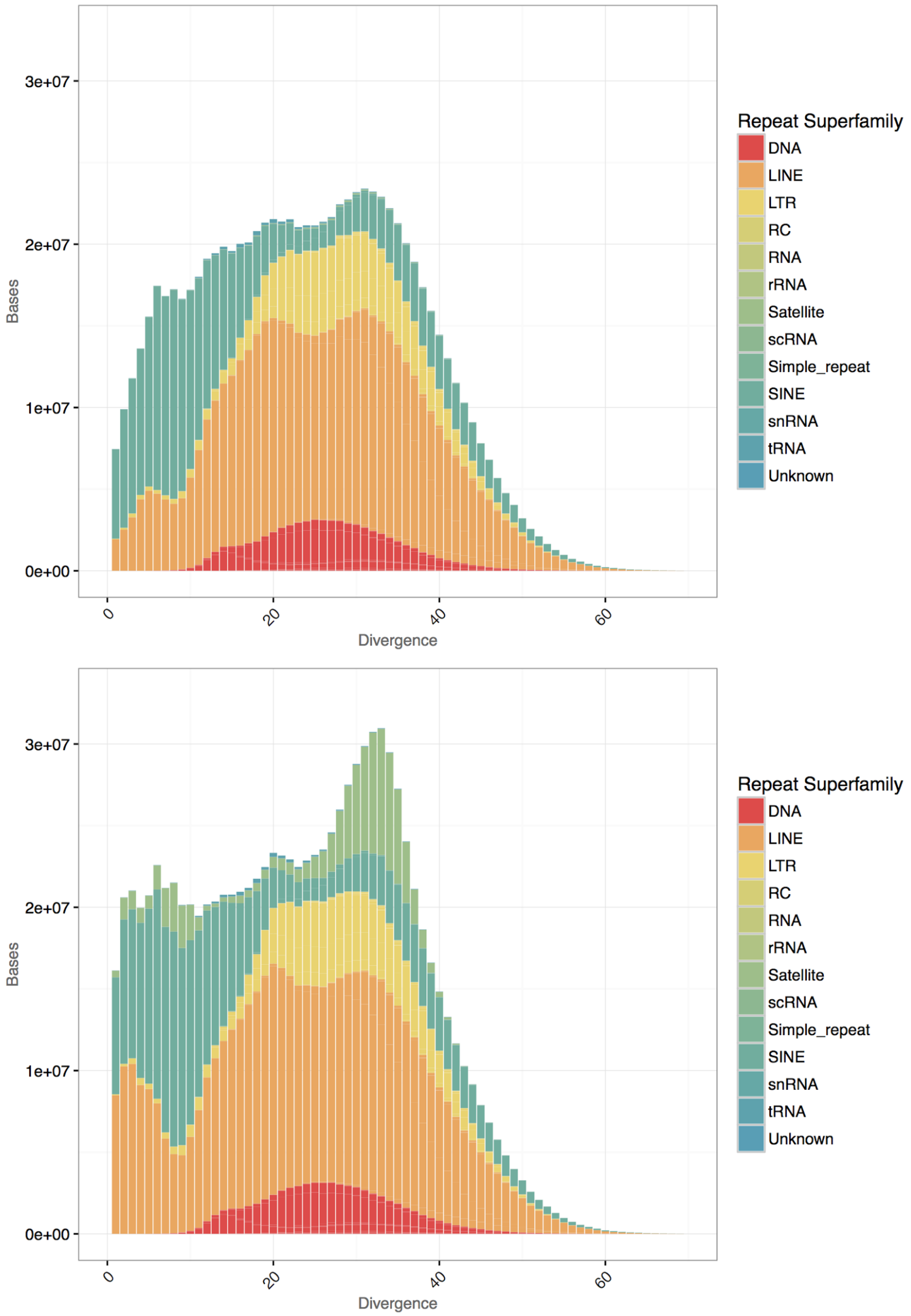

Supplementary Figure 3: Comparison of the divergence of the different repeat elements from their consensus sequence. The top panel shows the total number of bases against the divergence from the consensus sequence in each repeat family when using the de novo wolf

reference genome for alignment. The bottom panel shows the same figures when using the boxer reference genome.

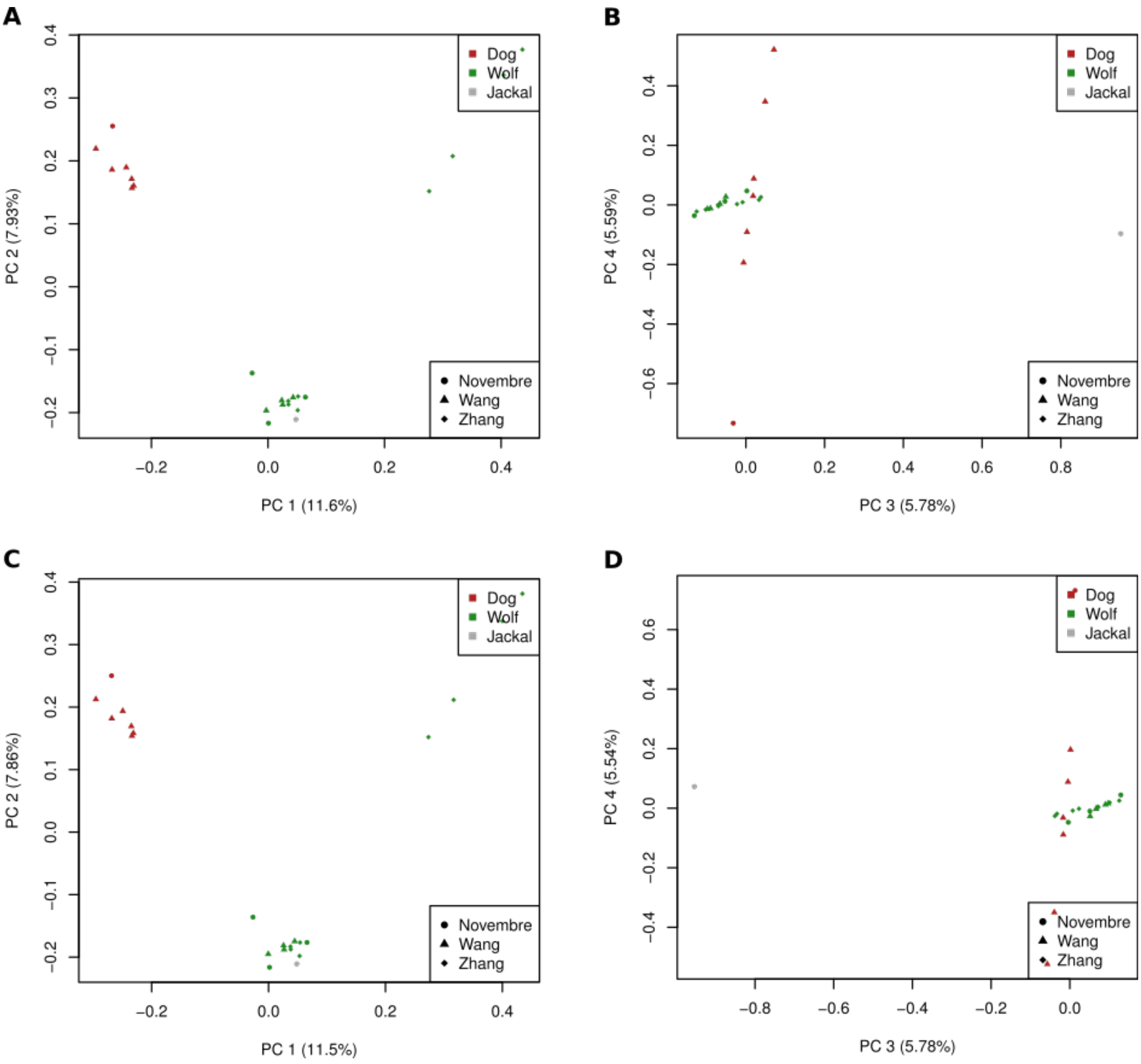

Supplementary Figure 4: Principal Components Analysis (PCA). Panels A and B show the first four principal components of the genotypes when using the de novo wolf reference assembly. For making these PCA plots, we used a missingness cutoff of 0.9 and a minor allele frequency cutoff of 0.2. Panels C and D show the first four principal components of the genotypes when using the boxer reference genome while using the same filtering thresholds.

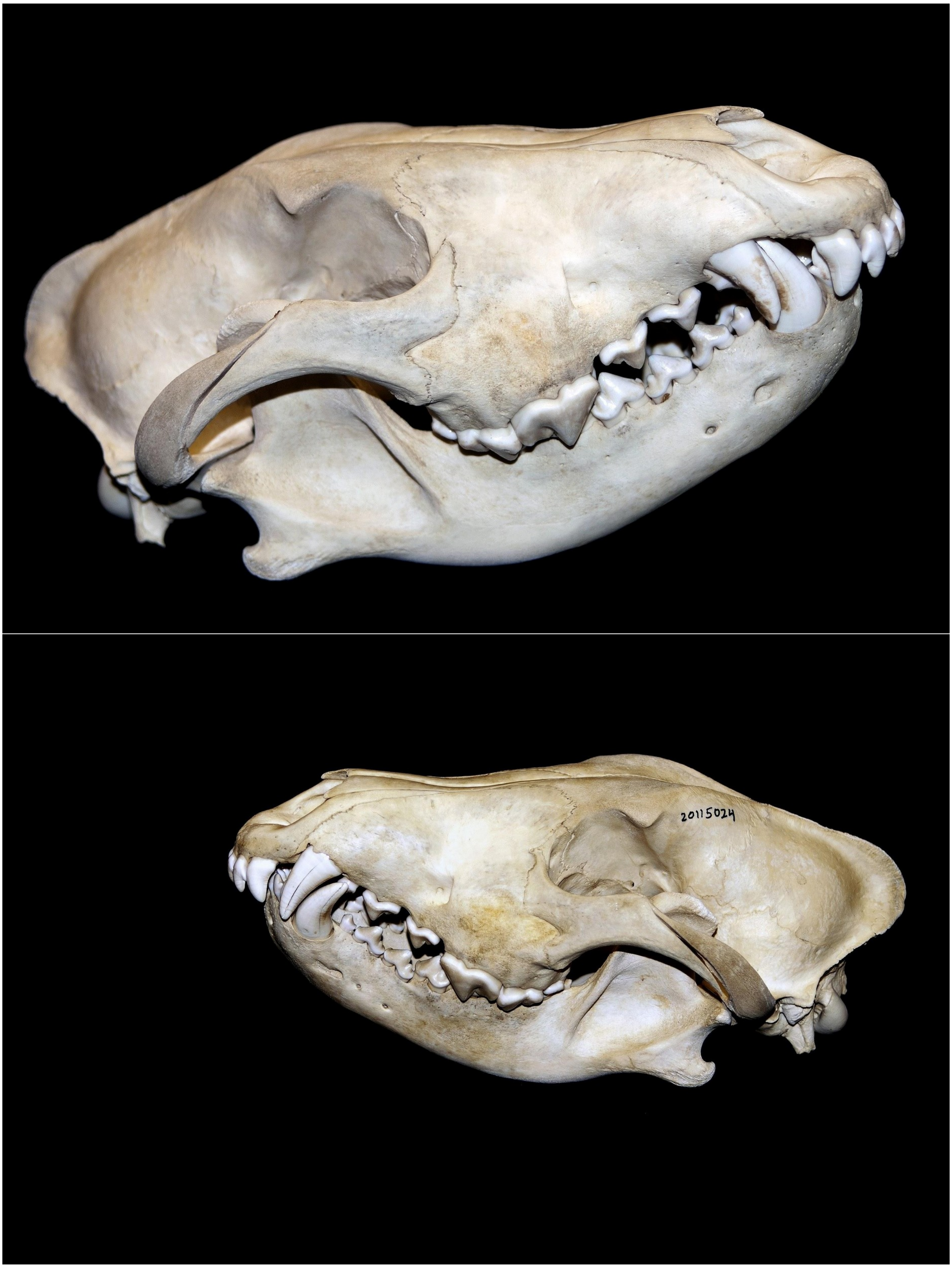

Supplementary Figure 5: Picture of the skull of the Swedish wolf sample used for reference genome assembly. Photo: J. Räikkönen

SUPPLEMENTARY TABLES

Study/<br>Sample	Aligned to the de novo wolf genome assembly		Aligned to the boxer dog reference genome	
	**Coverage**	**Heterozygosity**	**Coverage**	**Heterozygosity**
Freedman et al.				
Golden Jackal	21.996	0.00128457	**22.006**	0.000773261
Chinese Wolf	**25.477**	0.00119212	25.271	0.00126921
Croatian Wolf	**24.315**	0.00110828	24.157	0.000768382
Israeli Wolf	**20.620**	0.00135151	20.481	0.000883584
Dingo	25.440	0.000506813	**25.688**	0.000338711
Wang et al.				
Gray Wolf 1	**9.980**	0.00124735	9.932	0.0014045
Gray Wolf 2	**8.090**	0.00105924	8.047	0.00119143
Gray Wolf 3	**10.199**	0.00114766	10.169	0.00132054
Gray Wolf 4	**8.191**	0.000880498	8.163	0.000994077
Belgian Malinois	9.376	0.000714592	**9.419**	0.000795193
German Shepherd	8.582	0.000502106	**8.604**	0.000556685
Chinese Indigenous Dog 1	11.021	0.00105524	11.020	0.00117199
Chinese Indigenous Dog 2	9.917	0.000735783	**10.484**	0.000829596
Chinese Indigenous Dog 3	8.244	0.000867376	**8.25**	0.000965169
Tibetan Mastiff	9.478	0.000928	**9.516**	0.00104257
Zhang et al.				
Gray Wolf - C6	**23.133**	0.00130461	22.996	0.00145654
Gray Wolf - C7	**20.131**	0.00139529	20.022	0.00155539
Gray Wolf - C9A	**23.905**	0.000708302	23.767	0.000770862
Gray Wolf - C11	**23.705**	0.00131756	23.569	0.00146154
Gray Wolf - C16	**24.094**	0.00121058	23.952	0.00133022
Gray Wolf - C24	**21.797**	0.00151702	21.638	0.00169685
Gray Wolf - C30	**24.387**	0.00153458	24.213	0.00171929
Gray Wolf - C32	**23.068**	0.000834478	22.941	0.000919653

Supplementary Table 1: Coverage and heterozygosity estimates. The coverage and heterozygosity are shown for each sample included in the study. For each animal, the higher estimate of coverage are bolded.
